# Genetic modifiers of rare variants in monogenic developmental disorder loci

**DOI:** 10.1038/s41588-024-01710-0

**Published:** 2024-04-18

**Authors:** Rebecca Kingdom, Robin N. Beaumont, Andrew R. Wood, Michael N. Weedon, Caroline F. Wright

**Affiliations:** Department of Clinical and Biomedical Sciences, University of Exeter Medical School, Royal Devon & Exeter Hospital, Exeter, UK

**Keywords:** Genetics research, Genomics

## Abstract

Rare damaging variants in a large number of genes are known to cause monogenic developmental disorders (DDs) and have also been shown to cause milder subclinical phenotypes in population cohorts. Here, we show that carrying multiple (2−5) rare damaging variants across 599 dominant DD genes has an additive adverse effect on numerous cognitive and socioeconomic traits in UK Biobank, which can be partially counterbalanced by a higher educational attainment polygenic score (EA-PGS). Phenotypic deviators from expected EA-PGS could be partly explained by the enrichment or depletion of rare DD variants. Among carriers of rare DD variants, those with a DD-related clinical diagnosis had a substantially lower EA-PGS and more severe phenotype than those without a clinical diagnosis. Our results suggest that the overall burden of both rare and common variants can modify the expressivity of a phenotype, which may then influence whether an individual reaches the threshold for clinical disease.

## Abstract (web summary)

Analysis of genetic modifiers of 599 developmental disorder genes in the UK Biobank found that rare variant burden within this set, as well as the common polygenic background, can alter the expressivity of cognitive and socioeconomic traits in an additive manner.

## Main

Ascertaining whether rare genetic variants cause a monogenic phenotype can be challenging because of incomplete penetrance and variable expressivity^1^. Many rare variant studies use clinical or familial cohorts that can overestimate the penetrance of causal variants^2^. The presence of such rare, putatively damaging variants in healthy population cohorts^3^ can provide a lower boundary for estimates of penetrance, and individuals in both clinical and population cohorts display a spectrum of phenotypic variability caused by similar or identical variants^1,4^. Previous research has suggested that common genetic variants can modify the penetrance or expressivity of phenotypes caused by rare genetic variants^4–11^, potentially through the liability threshold model, which posits that a certain threshold of disease susceptibility needs to be crossed before clinically diagnosable disease manifests^11–14^. Some damaging rare variants may reach this threshold alone, resulting in a monogenic disease phenotype with 100% penetrance, whereas other variants may require additional genetic, environmental or other modifiers to reach this threshold^12^. In certain diseases, the common variant burden has been shown to confer a risk similar to that of a deleterious monogenic variant, where the highest polygenic risk may be equivalent to that conferred by a monogenic variant^15,16^. Because the effect of individual common variants is very small^17^, aggregating them together as a polygenic score (PGS) has become a widely used method for predicting overall risk^18,19^, and combining PGS with rare pathogenic variants could improve individual disease prediction^20,21^.

It has previously been shown that rare predicted loss-of-function (pLoF) variants, as well as deleterious missense and large copy number variants (CNVs), in genes and loci linked with severe monogenic developmental disorders (DDs) can have milder, subclinical effects in the general population^14,22–25^. The related common variant burden has been shown to affect the phenotype in carriers of such variants^5,26^, suggesting that the cumulative effect of common variants can modify the penetrance of rare variants in such phenotypes, even when the primary cause is considered monogenic. While the impact of common variants on overall phenotypic expressivity has been examined for several neuropsychiatric^25,27,28^ and other disease cohorts^29–31^, the modification of rare variant penetrance by other rare genetic variants has not been widely investigated because of the large cohort sizes required. Here, we present an analysis of common and rare variant burden in 419,854 adults from the UK Biobank (UKB)^32^. We investigated individuals carrying a rare pLoF variant in genes and loci where similar variants are known to cause monogenic DD and used related PGSs and additional rare variant burden to examine the effect on a number of related cognitive phenotypes and socioeconomic traits. We show that rare variant burden across these loci and an educational attainment (EA)-PGS have an additive effect on the phenotype. Our results demonstrate that both rare and common genetic variants linked to relevant traits can contribute to the variable expressivity of rare, predicted large-effect variants in known monogenic diseases.

## Results

We used exome sequencing and microarray data from individuals in UKB of genetically defined European ancestry (*n* = 419,854). We identified carriers of rare (allele count ≤ 5) pLoF^33^ or deleterious missense (REVEL > 0.7)^34^ variants in any of 599 genes from the Developmental Disorders Geneotype-to-Phenotype Database (DDG2P; Supplementary Table 1)^22,35^ in which damaging rare variants are a known cause of autosomal dominant DD. Carriers of multigenic CNVs were also included where the variant overlapped known syndromic DD-related loci^36,37^, as described previously^22^. We calculated the published EA-PGS^38^ using summary statistics and weighted allele effects from genome-wide association studies (GWAS) for every UKB individual of European ancestry. Phenotypes of interest were selected from self-reported questionnaires, based on their relevance to cognitive, behavioral, reproductive and socioeconomic traits related to neurodevelopmental disorders (Supplementary Table 2). In addition, clinically relevant diagnoses were identified using International Classification of Diseases (ICD)-9 or ICD-10 codes from hospital episode statistics and combined into three categories: (1) child DDs; (2) adult neuropsychiatric conditions (schizophrenia or bipolar disorder); and (3) other mental health issues (neurotic and anxiety disorders; Supplementary Table 3).

### Carrying multiple rare variants in monogenic DD loci is associated with an increased phenotype effect compared to single variant carriers

**Fig. 1:**
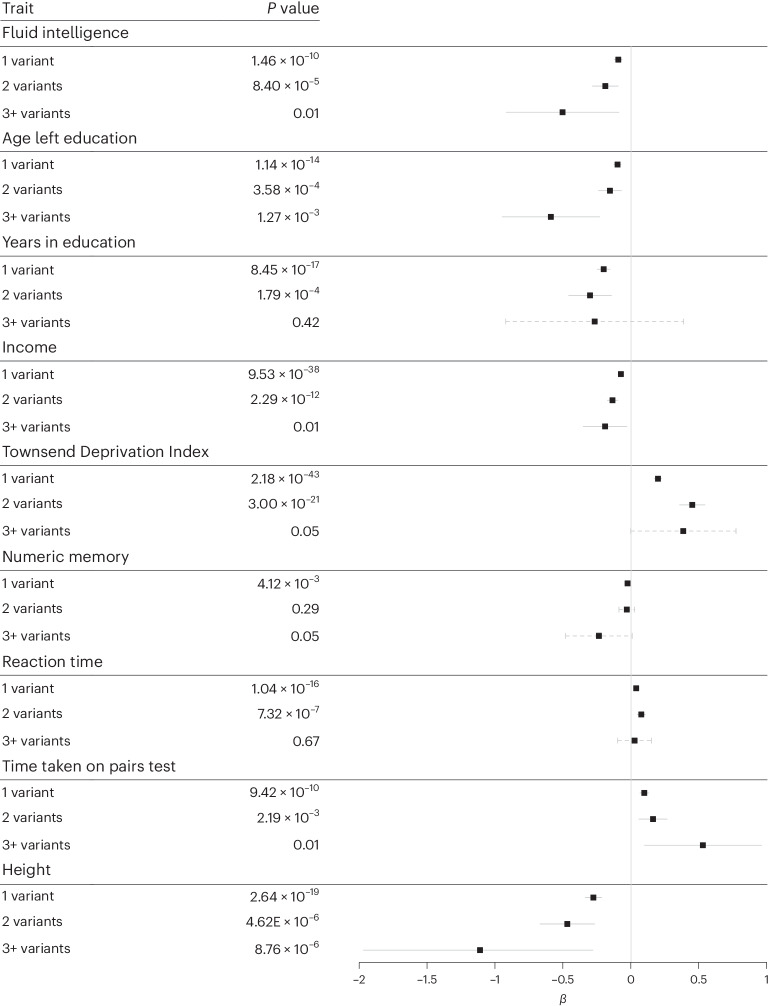
Effect of rare DD variant burden on continuous DD-related phenotypes in UKB. Linear regression of continuous traits in individuals carrying 1, 2 or 3+ rare pLoF, deleterious missense or multigenic variants overlapping dominant DDG2P genes, compared to the rest of UKB (that is, noncarriers). *β* values for continuous traits were measured as follows: fluid intelligence, standardized units (ranging from 1–13); age left education and years in education, years; height, cm; reaction time, time taken on pairs test, numeric memory, income, and TDI, standard deviations from the mean. Data are presented as mean values ±95% CI, where solid lines indicate that the *P* value reached Bonferroni-corrected significance and dashed lines indicate that it did not. *P* values were not corrected for multiple testing.

**Fig. 2:**
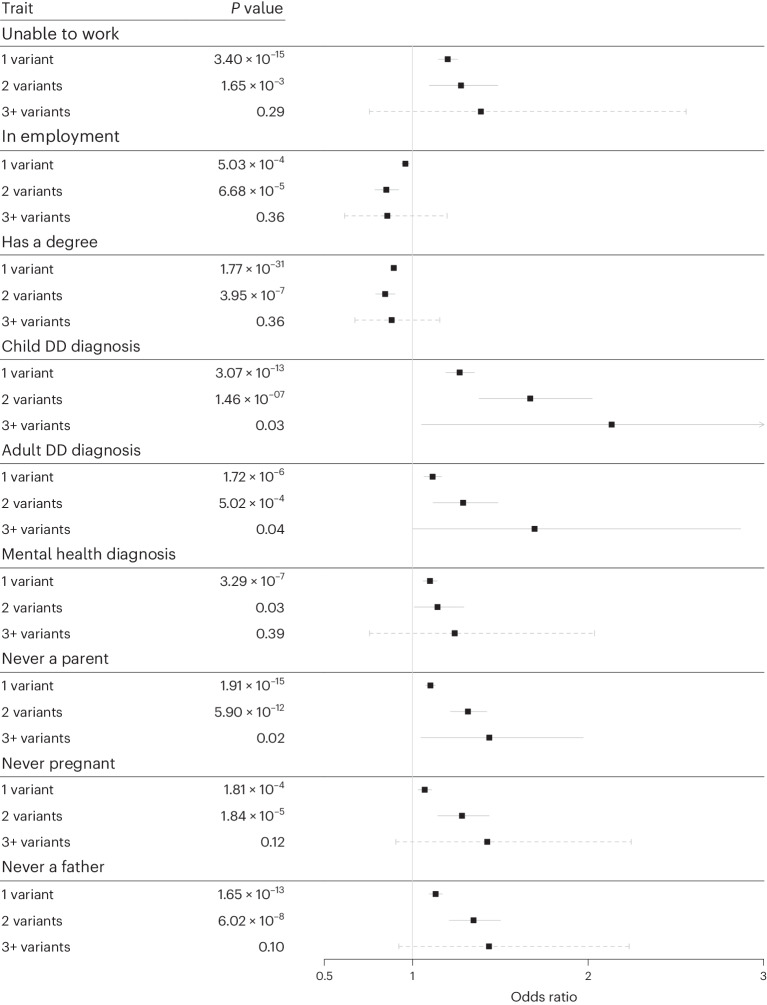
Effect of rare DD variant burden on binary DD-related phenotypes in UKB. Logistic regression of binary traits/diagnoses in individuals carrying 1, 2 or 3+ rare pLoF, deleterious missense or multigenic variants overlapping dominant DDG2P genes compared to the rest of UKB (that is, noncarriers). Data are presented as mean values ± 95% CI, where the solid lines indicate that the *P* value reached Bonferroni-corrected significance and the dashed lines indicate that it did not. *P* values were not corrected for multiple testing.

We first investigated whether DD-related phenotypes could be modified among rare DD variant carriers by the presence of additional rare pLoF or damaging missense variants in the same set of DDG2P genes. In UKB, 50,395 (12%) individuals carried a single rare, likely deleterious variant overlapping one of the 599 autosomal dominant DDG2P genes (12,153 pLoF and 35,603 missense) or syndromic DD loci (1,127 large deletions and 1,512 large duplications); an additional 3,831 individuals carried two rare DD variants and 219 individuals had three or more putatively deleterious rare variants across these DD loci. The highest overall rare variant burden across the DD loci was five, which was observed in two individuals with three missense variants and two pLoF variants each (Supplementary Table 4). We performed regression analyses to test associations between the number of rare variants in DD genes and 15 DD-related traits and diagnoses, using linear regression for continuous traits (Fig. 1) and logistic regression for binary traits (Fig. 2). Increasing rare variant burden was correlated with larger differences from the average UKB participant in several DD-related phenotypes, including lower fluid intelligence, shorter stature, lower income, lower likelihood of being employed, lower likelihood of being a parent and higher Townsend Deprivation Index (TDI). An increase in rare variant burden also correlated with a higher likelihood of having a DD-related diagnosis, and those with three or more rare DD variants were 2.1 times (95% confidence interval (CI), 1.05–4.33; *P* = 0.03) and 1.7 times (95% CI,1.01−2.89; *P* = 0.04) more likely to be diagnosed with a child DD or an adult DD-related neuropsychiatric disorder, respectively, than noncarriers (Fig. 2). When we excluded those with rare missense variants and considered only pLoF and large CNV carriers (Supplementary Table 5), we observed a larger change in phenotype, but the smaller number of individuals present in each group substantially reduced the statistical power; nonetheless, those with two or three rare variants were 2.2 times (95% CI,1.37–3.43; *P* = 0.0009) more likely to have a child DD-related diagnosis than those without a pLoF variant or CNV.

### Polygenic background modifies the phenotype of carriers of rare variants in monogenic developmental disorder loci

**Fig. 3:**
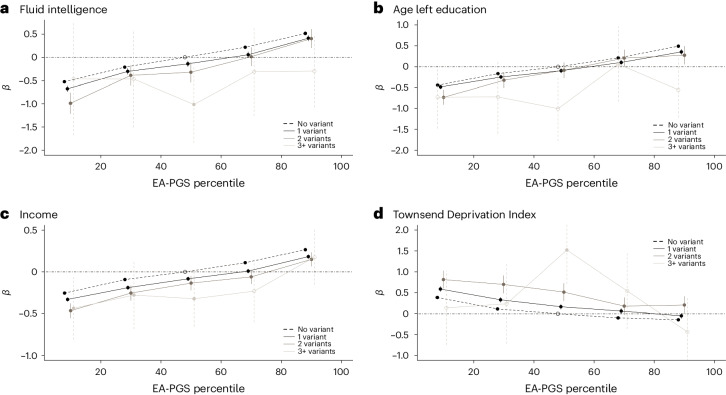
Additive effect of rare DD variant burden and EA-PGS on DD-related phenotypes. **a**−**d**, Linear regressions of fluid intelligence (**a**), age left education (**b**), income (**c**) and TDI (**d**) versus the EA-PGS quintile in UKB. The black dashed horizontal line corresponds to noncarriers of rare DD variants (*n* = 365,409); dark/medium/light horizontal lines correspond to carriers of 1, 2 or 3+ rare DD variants (*n* = 50,395, 3,831 and 219), respectively. Notably, within UKB, a sufficiently high EA-PGS can compensate for the effect of a primary variant and, in most cases, any additional rare DD variants on these traits. Data are presented as mean values ± 95% CI (vertical lines), where solid vertical lines indicate that the *P* value reached Bonferroni-corrected significance and dashed vertical lines indicate that it did not. *P* values were not corrected for multiple testing.

Next, we investigated the effect of common polygenic background on rare DD variant carriers^13^. We separated the UKB cohort into five EA-PGS quintiles and repeated the phenotype association tests with rare DD variant carrier status. We observed a similar trend across all traits tested against the EA-PGS quintiles (Supplementary Fig. 1), with the direction of the PGS effect being the same in both carrier and noncarrier groups. Individuals who carried at least one rare variant showed a consistently larger change in fluid intelligence, years of education, employment status and TDI across the PGS spectrum compared to the control group, with larger phenotypic effects observed in carriers of multiple rare DD variants (Fig. 3). We observed similar trends when we repeated this analysis using an earlier GWAS of EA that excluded UKB^39^ (Supplementary Fig. 2) and for GWAS of intelligence^40^ (Supplementary Fig. 3) and cognitive or mathematical abilities^17^ (Supplementary Fig. 4), as well as when excluding missense variants (Supplementary Table 6), or using a smaller subset of DD genes (Supplementary Table 7) known to cause disease via haploinsufficiency (*n* = 325) or only those that reached genome-wide significance based on the burden of de novo variants in ~31,000 DD cases (*n* = 125)^41^.

For fluid intelligence, the difference in the mean score between the bottom and top EA-PGS quintiles equated to approximately 1 point on the 13-point scale (approximately 0.5 s.d.), for both rare variant carriers and noncarriers in UKB. Rare DD variant carrier status was equivalent to approximately a 20-percentile-point decrease in EA-PGS, on average, with the result that an EA-PGS above the 70th percentile was able to compensate for the effect of carrying a single rare DD variant on fluid intelligence (Supplementary Table 8). Rare variant carrier status and EA-PGS appeared to have an additive effect when assessed against multiple related traits, with the effect of rare variants remaining similar throughout the EA-PGS spectrum. When we investigated rare variant classes within fluid intelligence scores, deleterious missense variant carriers reached parity with the control group at the 62nd EA-PGS percentile, pLoF carriers at the 80th percentile and CNV duplication carriers at the 82nd percentile, whereas CNV deletion carriers never reached parity with the control group (Supplementary Table 8).

We were interested in exploring whether there was an enrichment of DDG2P genes in EA GWAS loci. We hypothesized that the EA-PGS could include single-nucleotide polymorphisms (SNPs) in *cis*-regulatory regions of monogenic DDG2P genes; therefore, we examined the proximity between the 599 autosomal dominant DDG2P genes and 3,952 SNPs included in the EA-PGS, using simulations of matched SNPs (10,000 lists of matched SNPs per GWAS SNP, based on allele frequency and proximity to genes) to empirically test whether the genes fall disproportionately close to the GWAS loci^42^. As expected, we found that the GWAS loci were closer to DDG2P genes than expected by chance alone (*P* = 0.005), suggesting that the large-effect rare variants and small-effect common variants may work through overlapping biological pathways.

As the UKB cohort is known to be biased toward healthier, wealthier and more educated individuals than the general population^43^, we hypothesized that individuals in UKB who carry a rare DD variant might also have a higher EA-PGS on average than the noncarrier control group, which partially compensates for the potentially deleterious effects of the rare DD variant. Overall, we observed that individuals who carried at least one rare DD variant did indeed have a slightly higher EA-PGS percentile than noncarriers (two-sided *t*-test difference = +2.1; 95% CI, 1.9–2.4; *P* < 0.0005), supporting this hypothesis. Furthermore, among the small number of individuals who achieved the top score on the fluid intelligence test (*n* = 139), we observed that rare DD variant carriers (*n* = 4) were depleted versus the rest of UKB participants (3% versus 13%; *P* = 0.0002) and had a substantially higher EA-PGS percentile than noncarriers (two-sided *t*-test difference = +26.1; 95% CI, 1.8–50.3; *P* = 0.04).

### Rare variant status and polygenic background additively contribute to phenotype and predict outliers

**Fig. 4:**
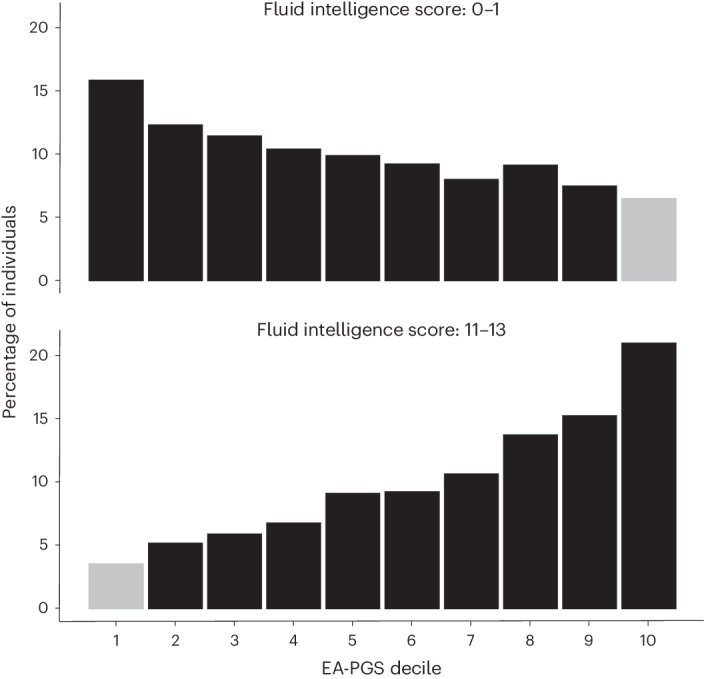
Distribution of EA-PGS and fluid intelligence within UKB. Phenotypic deviators are highlighted and defined as either individuals in a top EA-PGS decile with a low fluid intelligence score (0 or 1) or those in a bottom EA-PGS decile with a high fluid intelligence score (11, 12 or 13). All individuals (*n* = 419,854) were included.

**Fig. 5:**
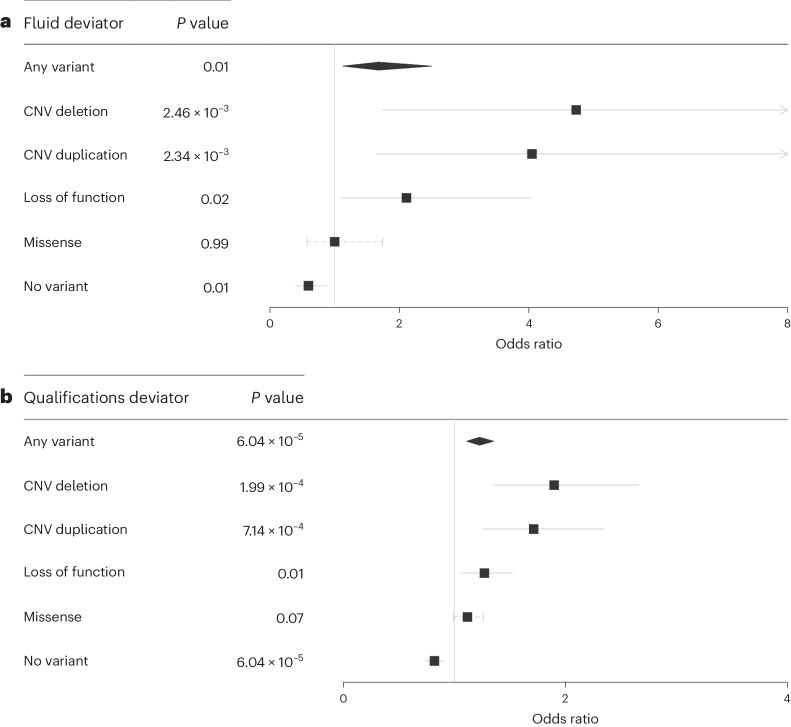
Rare DD variant carrier status of phenotypic deviators from EA-PGS predictions. **a**,**b**, Logistic regression showing that individuals in UKB who either had an EA-PGS in the top decile but scored low on the fluid intelligence test (*n* = 137) (**a**) or reported having no qualifications recorded despite having an EA-PGS in the top decile (*n* = 4,292) (**b**) were more likely to be rare DD variant carriers. The comparator group included those within the same EA-PGS decile but with a higher fluid intelligence score or recorded qualifications. Data are presented as mean values ± 95% CI, where solid lines indicate that the *P* value reached Bonferroni-corrected significance and the dashed lines indicate that it did not. *P* values were not corrected for multiple testing.

Intrigued by the presence of these apparently highly intelligent rare DD variant carriers, we further investigated phenotypic ‘deviators’ in whom the predicted genetic susceptibility was discordant with the observed phenotype^44^, for example, individuals with high EA-PGS but low fluid intelligence score and vice versa (Fig. 4). This question has particular clinical relevance as it has previously been suggested that individuals with familial disease could be prioritized for genetic testing based on having a low-risk PGS because they may be more likely to have a single large-effect causal variant than individuals with a high-risk PGS whose disease could be more polygenic^45,46^. To investigate this hypothesis, we further split the UKB cohort into EA-PGS deciles and tested whether individuals whose low cognitive phenotype was discordant with their high EA-PGS were more likely to be rare DD variant carriers than the remainder of the UKB cohort. Individuals in the top EA-PGS decile but with low fluid intelligence (scores of 0 or 1 of 13) were more likely to be rare DD variant carriers (odds ratio (OR) = 1.68; 95% CI, 1.13–2.50; *P* = 0.01) (Fig. 5a) when compared to those in the same EA-PGS decile who did not have a low fluid intelligence score, as were those in the top EA-PGS decile who had no educational qualifications on record (OR = 1.22; 95% CI, 1.10–1.35; *P* = 0.00006) (Fig. 5b). Following separation by rare DD variant class, we found that large multigenic deletions had a larger effect than any other type of rare DD variant (OR = 4.7; 95% CI, 1.73–12.95; *P* = 0.002), followed by multigenic duplications and then by pLoF variants (Supplementary Table 9). We then investigated whether the opposite was also true, that is, whether those with an EA-PGS in the bottom decile but a high fluid intelligence score (11–13 of 13) were less likely to be rare variant carriers, and found that these individuals were nearly half as likely as others in the same decile to carry a rare DD variant (OR = 0.58; 95% CI, 0.38–0.87; *P* = 0.009).

**Fig. 6:**
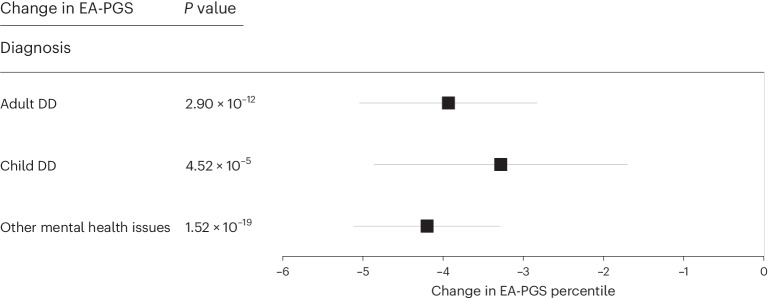
Average change in EA-PGS among rare DD variant carriers with a relevant clinical diagnosis. Linear regressions among individuals carrying one or more rare DD variants, including those who were clinically diagnosed with child DDs (*n* = 7,933), adult neuropsychiatric conditions (*n* = 19,004) or other mental health issues (*n* = 32,911), with EA-PGS, showing that those with a clinical diagnosis have a substantially lower EA-PGS than those who do not have a related clinical diagnosis recorded in UKB. Data are presented as mean values ± 95% CI, where solid lines indicate that the *P* value reached Bonferroni-corrected significance and dashed lines indicate that it did not. *P* values were not corrected for multiple testing.

Finally, we investigated whether a decrease in EA-PGS correlated with the likelihood of receiving a clinical diagnosis related to DD among the rare DD variant carriers identified in UKB. The number of individuals identified within the three diagnostic categories (child DDs, *n* = 7,933; adult neuropsychiatric conditions, *n* = 19,004; and other mental health issues, *n* = 32,911) is likely to be an underestimate because of missing data or the absence of, or omissions in, individual hospital records available within UKB. Therefore, although individuals in any of these diagnostic categories were more likely to be rare DD variant carriers than the rest of UKB, the majority did not carry a rare variant in any of the DD genes, and many individuals with a rare DD variant did not have a corresponding diagnosis. Despite these limitations, we found that, among rare DD variant carriers, those with a related clinical diagnosis across any of our three categories had a substantially lower EA-PGS than those without a diagnosis (Fig. 6); rare DD variant carriers with adult neuropsychiatric disorders or mental health issues (but not child DDs) also had a higher schizophrenia or bipolar PGS (Supplementary Fig. 5). Rare DD variant carriers with a diagnosis also had a larger phenotypic change than other rare variant carriers without a diagnosis; individuals with a rare DD variant and a related clinical diagnosis were more likely to be unable to work (OR = 6.66; 95% CI, 6.07–7.32; *P* = 4.51 ⨯ 10^−308^), less likely to have a degree (OR = 0.71; 95% CI, 0.66–0.76; *P* = 3.76 ⨯ 10^−23^) and less likely to be employed (OR = 0.33; 95% CI, 0.31–0.37; *P* = 2.07 ⨯ 10^−143^) than those who carried a rare DD variant but did not have a diagnosis recorded in UKB (Supplementary Table 10). This suggests that both the aggregation of the overall number of rare DD variants carried and a lower EA-PGS can alter the overall expressivity of the phenotype toward reaching the threshold of clinical disease.

## Discussion

We showed that the phenotypic effect of a heterogeneous set of rare disease-associated variants is modified by both additional rare and common genetic variants in a population cohort. The adverse effects of carrying a single rare deleterious variant in genes in which similar variants cause monogenic DD can be modified by additional rare variants in those genes or by common variants across the genome. We found that carriers of multiple rare DD variants in UKB have lower fluid intelligence, shorter stature, fewer children, lower income, higher unemployment and a higher TDI than carriers of single rare DD variants. In addition, our results suggest that having a higher EA-PGS can partially compensate for the negative cognitive and socioeconomic effects of carrying either a single or multiple rare DD variants. Moreover, an increased burden of DD-associated variants is more likely to shift the phenotypic presentations over the threshold for clinical diagnosis and correlates with a greater change in phenotype compared to individuals who carry fewer or no variants. Our results suggest that the PGS may provide some clinical utility by improving the diagnostic interpretation of rare, likely pathogenic variants that cause monogenic disease.

Investigating the effect of pathogenic rare variants in the general population is important for understanding the penetrance and variable expressivity of monogenic diseases. We have shown that approximately 12% of UKB participants carry a rare predicted damaging variant in one of 599 dominant DD genes (5%, excluding missense), and a further 1% carry a rare predicted damaging variant in more than one of these genes (0.1%, excluding missense), conferring a higher risk of impaired cognitive performance and neuropsychiatric conditions. However, there are important limitations to the use of large-scale genetic data from UKB to investigate rare diseases. First, some of the deleterious rare variants we identified may be benign, due to technical artifacts, or erroneous pathogenicity predictions, or be rescued by alternative splicing or other molecular mechanisms. Second, UKB is known to have an ascertainment bias toward healthier and wealthier individuals compared to the rest of the British population^43^, and individuals affected by severe, highly penetrant monogenic disorders are likely to be underrepresented in the cohort. Third, because UKB is a relatively old cohort, complete medical histories are not always available, and therefore, many phenotypes of relevance to childhood DDs cannot be evaluated. Fourth, environmental influences were not assessed and yet these influences may have additional effects on the overall phenotype^47,48^ and could alter the penetrance and expressivity of genetic variants through gene−environment interactions. Finally, there are challenges in applying common variant PGSs across a population, as the underlying summary statistics are heavily dependent upon the populations and ethnicities in which the GWAS were performed. While it would have been optimal to use a PGS derived independently of UKB, we chose to use the largest and most recent EA-PGS from Okbay et al.^38^, in which UKB constitutes only a small part of the GWAS discovery cohort (~15% of the total of >3 million individuals). Given the small overlap and large sample size, it is unlikely that using this EA-PGS would result in substantial overfitting in UKB. Importantly, our results are consistent with those of previous studies showing the effect of rare DD variants in nonclinical cohorts and the modifying effect of the PGS on carriers of rare DD variants^5,6^.

In conclusion, we have shown that common and rare genetic variants can additively and independently affect the phenotype of nonclinically ascertained individuals. Our results help to explain the puzzling observation of apparently healthy carriers of monogenic likely disease-causing variants in the general population, as well as instances of incomplete penetrance and variable expressivity in families affected by rare diseases. Further research is needed to investigate other modifiers, such as rare noncoding variants and gene−environment interactions, and to understand the mechanisms by which genetic modifiers act. Ultimately, incorporating the additive effects of both rare and common variants will improve our understanding of disease.

## Methods

The UKB resource was approved by the UK Biobank Research Ethics Committee and all participants provided written informed consent to participate. This research was conducted using the UK Biobank resource under application numbers 49847 and 9072.

### UKB cohort

UKB is a voluntary population-based cohort from the UK with deep phenotyping data and genetic data for approximately 500,000 individuals aged 40–70 years at recruitment (54% female). Individuals provided various information via self-report questionnaires, and additional information was obtained from cognitive and anthropometric measurements and hospital episode statistics, including ICD-9 and ICD-10 codes. Genotypes of SNPs were generated using the UKB Axiom array (Affymetrix, ~450,000 individuals) and the UK BiLEVE array (~50,000 individuals). This dataset underwent extensive central quality control (http://biobank.ctsu.ox.ac.uk). A subset of the ~450,000 individuals from the UKB array also underwent exome sequencing using the IDT xGen Exome Research Panel v1.0 and this dataset was made available for research in October 2021 (ref. ^32^). Detailed sequencing and variant detection methodology for UKB is available at https://biobank.ctsu.ox.ac.uk/showcase/label.cgi?id=170. In brief, sequencing data were aligned to GRCh38 and variants were called using GATK 3.0 with hard filtering of variants with inbreeding coefficients < −0.03 or without at least one variant genotype of DP ≥ 10, GQ ≥ 20 and, if heterozygous, AB ≥ 0.20. We restricted our statistical analyses to 419,854 individuals with genetically defined European ancestry. European ancestry was defined by performing principal component analysis in the 1000 Genomes project reference panel using a subset of variants that were of high quality in UKB participants. We then used these loadings to project all UKB samples into the same principal component space and used a *k*-means clustering approach to define a European cluster using principal components 1–4.

### Gene selection

We used the clinically curated DDG2P to select genes known to cause monogenic DD. The database (accessed from https://www.ebi.ac.uk/gene2phenotype/ on 27 November 2020) was constructed and clinically curated from published literature and provides information relating to genes, variants and phenotypes associated with DDs, including the mode of inheritance and mechanism of pathogenicity. We included all genes that had been annotated as monoallelic (that is, autosomal dominant) with an evidence level of ‘confirmed’ or ‘probable’ (*n* = 599).

### Variant selection

We used exome sequencing data from 419,854 individuals in UKB to identify carriers of rare SNVs and/or insertions/deletions (indels) in any of the selected DDG2P genes. For our analyses, rare was defined as any variant that occurred in five or fewer individuals in the UKB cohort, excluding any variants with read depth <10⨯ or variant allele fraction <0.3. We selected two functional classes of variants in canonical transcripts based on annotation by the Ensembl Variant Effect Predictor (v104)^35^: (1) likely deleterious loss-of-function variants, defined as variants predicted to cause a premature stop, a frameshift or to abolish a canonical splice site; only those variants outside of the last exon and deemed to be high confidence by the Loss-Of-Function Transcript Effect Estimator (LOFTEE) were retained (https://github.com/konradjk/loftee); and (2) likely deleterious missense variants, defined as missense variants with a REVEL score >0.7. Individuals with >1 variant within a 40-bp window in the same gene were counted once. In addition, we used SNP array data from 488,377 genotyped individuals in UKB and PennCNV^49^ (v1.0.4) to detect multigenic CNVs that overlapped with 69 published CNVs strongly associated with developmental delay, as described previously^22^.

### PGS calculations

We created the EA-PGS using GWAS summary statistics from a large cohort meta-analysis, using 3,952 SNPs for the EA-PGS, with data from Okbay et al.^38^. The EA-PGS was calculated as ∑_*i*_*w*_*i*_*g*_*i*_, where *w*_*i*_ is the weight (effect size) of SNP *i* and *g*_*i*_ is the genotype (number of effect alleles, 0–2) of SNP *i*. The SNP weightings were the regression coefficients obtained from the most recently reported GWAS as mentioned above. We performed a sensitivity analysis using a PGS derived from 74 SNPs associated with EA in an earlier GWAS from Okbay et al.^39^, which excluded UKB (Supplementary Fig. 2). Other PGSs were similarly calculated from GWAS of intelligence^40^ and cognitive ability^17^, and we used PGSs released by UKB for schizophrenia and bipolar disorder^18^.

### Phenotype selection

We included the following phenotypes based on self-reported questionnaires and hospital episode statistics:

Mental health: a mental health issue was self-reported through a questionnaire or by ICD-10 codes F40−F48, F50, F51, F53, F54, F99, G47 and R45 or ICD-9 codes 300, 307–309, 311 and 780.5.

Diagnosed with ‘child DD’: intellectual disability (ICD-10 codes F70−F73), epilepsy (G40), developmental disorders (F80−F84, F88−F95, F98, R62, R48 and Z55) and congenital malformations (Q0−Q99).

Diagnosed with an ‘adult neuropsychiatric’ condition: including schizophrenia (self-reported or ICD-10 codes F20−F29) and bipolar disorder (self-reported or ICD-10 codes F30−F39).

Reproductive: never a parent, never a father or never pregnant.

Physical: height.

Cognitive: fluid intelligence (field ID: 20016), reaction time (inverse normalized, field ID: 20023), time to complete the pairs matching test (averaged, field ID: 20133), numeric memory (inverse normalized, field ID: 20240), age left education, number of years of education and had a degree.

Socioeconomic: employed, not able to work (both field ID: 6142), income (field ID: 738) and TDI (field ID: 189).

### Statistical analysis

We performed gene panel burden tests across our 599-gene subset, with association tests limited to individuals in UKB with genetically defined European ancestry because of well-recognized biases in PGS performance in other ancestries^50^. All analyses were controlled for age, sex, recruitment center and 40 principal components. Variant burden tests were performed using STATA (v16.0), using linear regression for continuous phenotypes and logistic regression for binary phenotypes with a Bonferroni-corrected *P* value of 0.05/18 = 0.003. Associations were tested between individuals with an identified rare variant in any of the DDG2P genes and the remainder of the European UKB population. EA-PGS quintiles were defined using the entire cohort of European UKB participants. When testing across PGS quintiles, each group was tested against individuals in the middle quintile (that is, those with a 40–60% EA-PGS) who were not identified as carriers of likely deleterious rare variants in the DDG2P gene subset. When testing associations within specific types of variants, the comparison group similarly included those not identified as carriers of likely deleterious variants. When testing smaller subgroups of individuals, the individuals previously identified as putatively deleterious variant carriers were removed from the comparison group. To define phenotypic deviators, we used the highest and lowest fluid intelligence scores (0 and 1 versus 11, 12 and 13) and the top and bottom categories for qualifications (no qualifications recorded versus having a degree).

### Reporting summary

Further information on research design is available in the Nature Portfolio Reporting Summary linked to this article.

## Online content

Any methods, additional references, Nature Portfolio reporting summaries, source data, extended data, supplementary information, acknowledgements, peer review information; details of author contributions and competing interests; and statements of data and code availability are available at 10.1038/s41588-024-01710-0.

### Supplementary information

Supplementary Information Supplementary Figs. 1−5.

Reporting Summary

Supplementary Tables Supplementary Tables 1−10.

Supplementary Data STATA scripts.

## Data Availability

The UK Biobank data are publicly available to approved researchers at https://biobank.ndph.ox.ac.uk/showcase/. The list of genes used for the analyses described in this paper are included in Supplementary Table 1, and the updated versions of DDG2P can be downloaded at https://www.ebi.ac.uk/gene2phenotype/.

## Code availability

STATA scripts are available as Supplementary Data.
