# Supplementary material for: Genetic modifiers of rare variants in monogenic developmental disorder loci
Source: Nat Genet. 2024 Apr 18;56(5):861–8. doi: 10.1038/s41588-024-01710-0 (PMC11096126; doi:10.1038/s41588-024-01710-0)
Supplement: Supplementary file 1 — Supplementary Figs. 1−5. [file 41588_2024_1710_MOESM1_ESM.pdf]

nature genetics

Article

https://doi.org/10.1038/s41588-024-01710-0

Genetic modifiers of rare variants in monogenic developmental disorder loci

In the format provided by the authors and unedited

SUPPLEMENTARY INFORMATION

Table of contents

Supplementary Fig. 1: Trait results across EA-PGS quintiles for variant carriers and non-carriers. 2

Supplementary Fig. 2: Sensitivity analysis of the additive effect of rare DD variant burden and EA-PGS on DD-related phenotypes. 3

Supplementary Fig. 3: Effect of rare DD variant burden and intelligence PGS on DD-related phenotypes. 4

Supplementary Fig. 4: Effect of rare DD variant burden and cognitive ability PGS on DD-related phenotypes. 5

Supplementary Fig. 5: Average change in schizophrenia PGS and bipolar disorder PGS among rare DD variant carriers with a relevant clinical diagnosis. 6

Supplementary References 7

Supplementary Fig. 1: Trait results across EA-PGS quintiles for variant carriers and non-carriers. Data are presented as mean values +/- 95% confidence intervals.

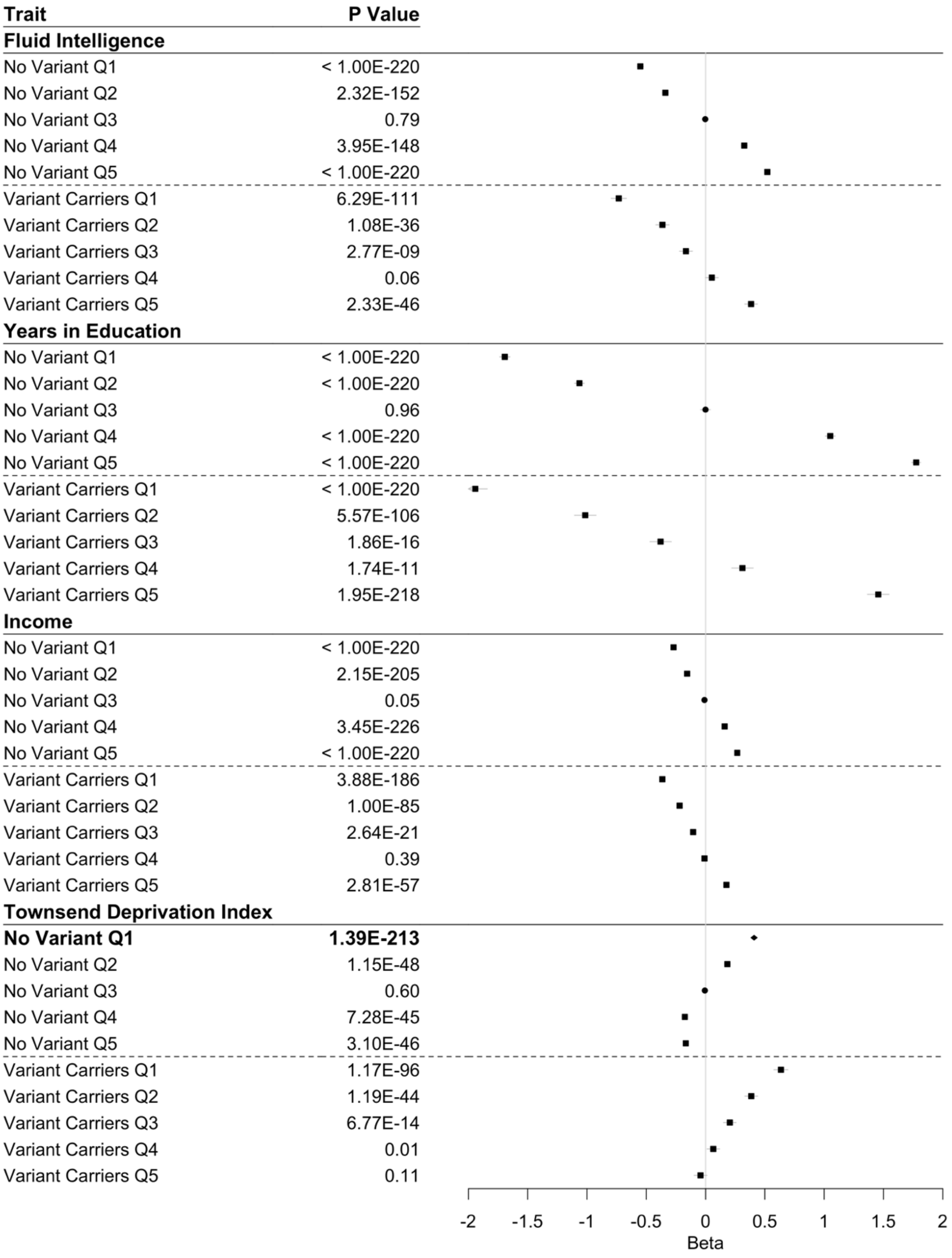

Supplementary Fig. 2: Sensitivity analysis of the additive effect of rare DD variant burden and EA-PGS on DD-related phenotypes.

This analysis used 74 SNPs from an EA GWAS that excluded UKB, Okbay *et al.* 2016[1]. Data are presented as mean values +/- 95% confidence intervals.

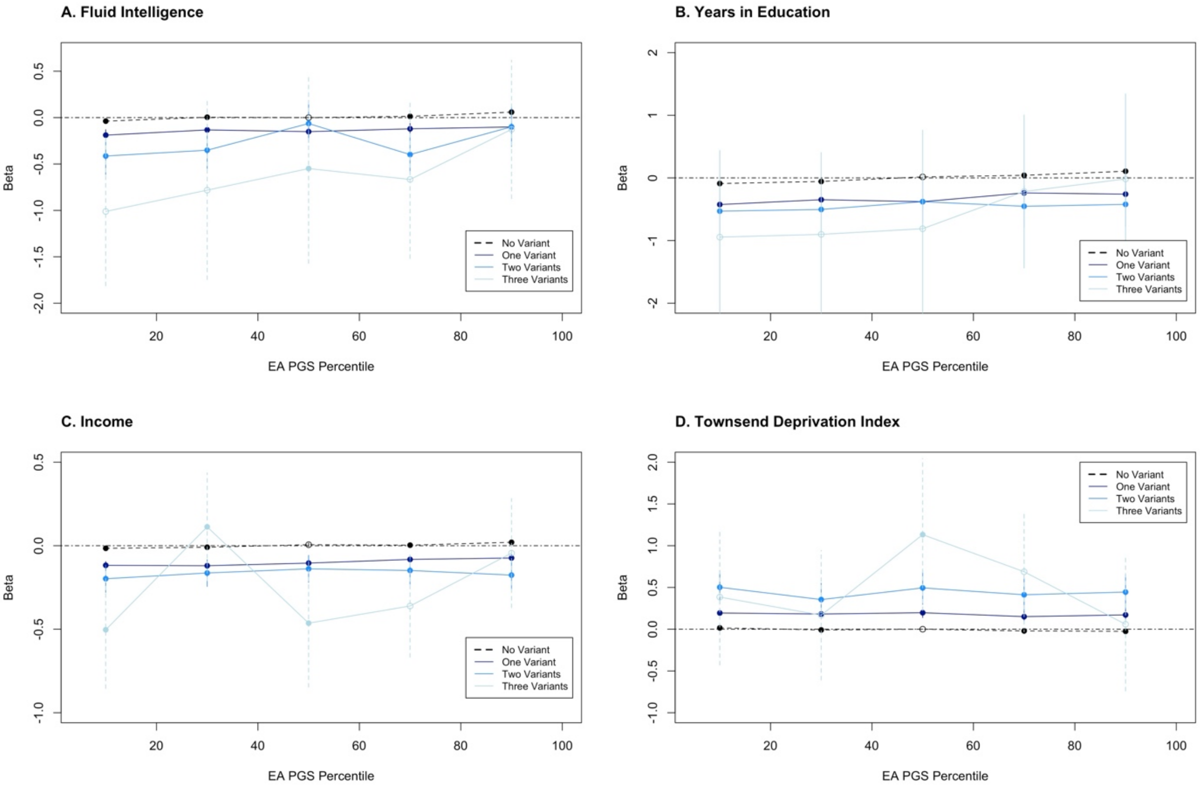

Supplementary Fig. 3: Effect of rare DD variant burden and intelligence PGS on DD-related phenotypes.
Intelligence PGS is derived from Savage *et al.* 2018[2] and Data are presented as mean values +/- 95% confidence intervals.

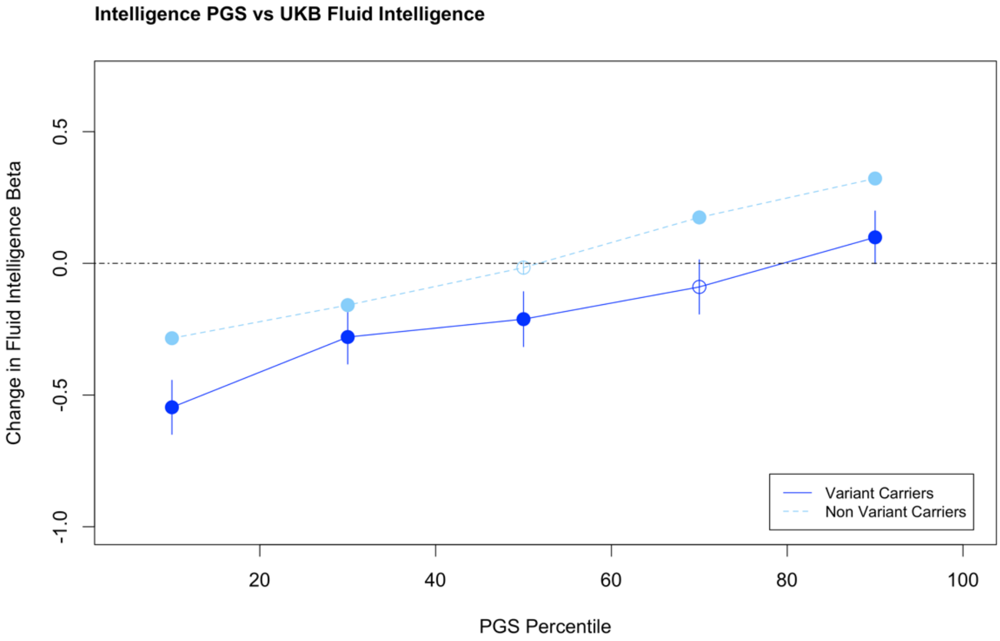

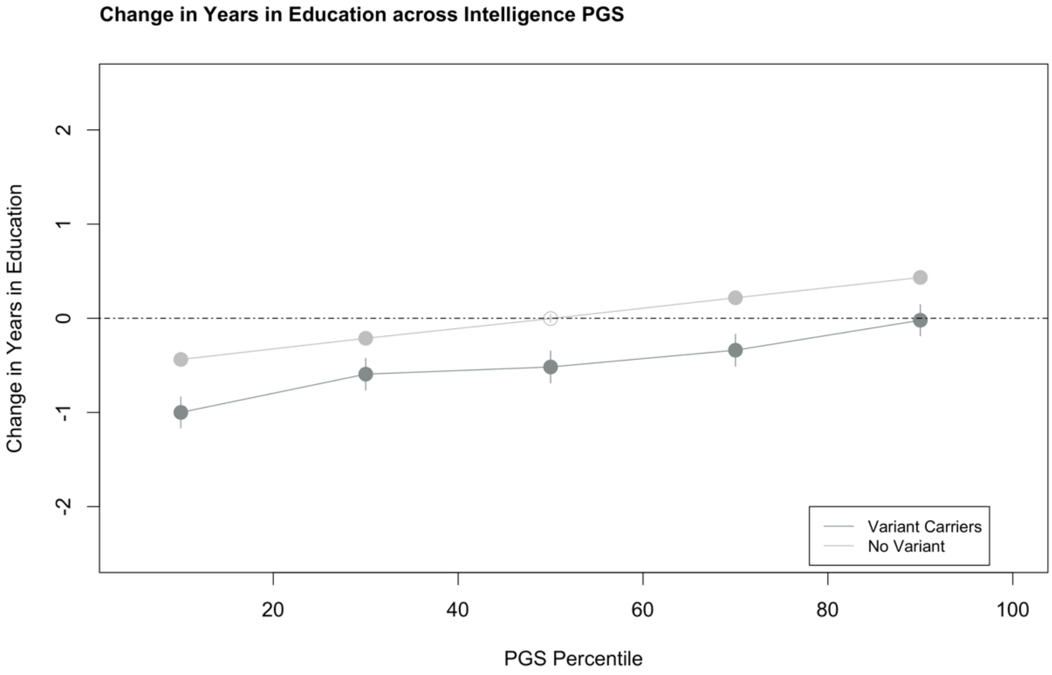

Supplementary Fig. 4: Effect of rare DD variant burden and cognitive ability PGS on DD-related phenotypes.

Cognitive ability PGS is derived Genç *et al* 2021[3]. Data are presented as mean values +/- 95% confidence intervals.

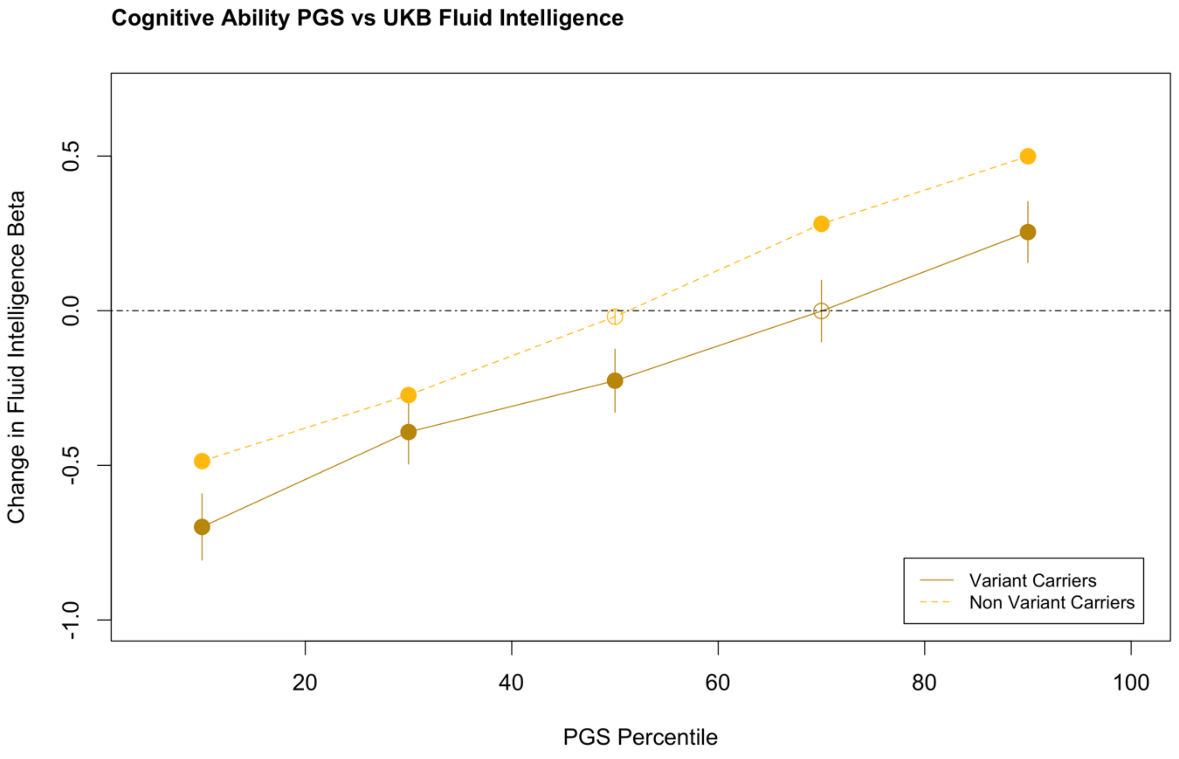

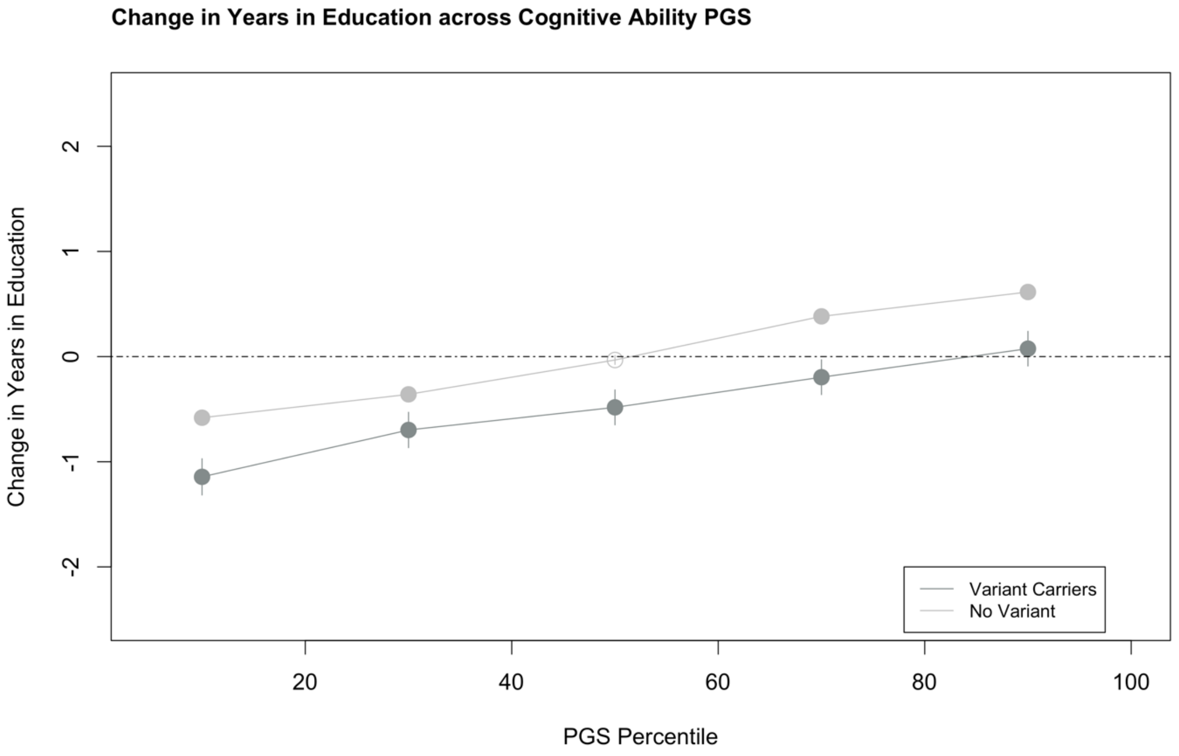

Supplementary Fig. 5: Average change in schizophrenia PGS and bipolar disorder PGS among rare DD variant carriers with a relevant clinical diagnosis.
Data are presented as mean values +/- 95% confidence intervals.

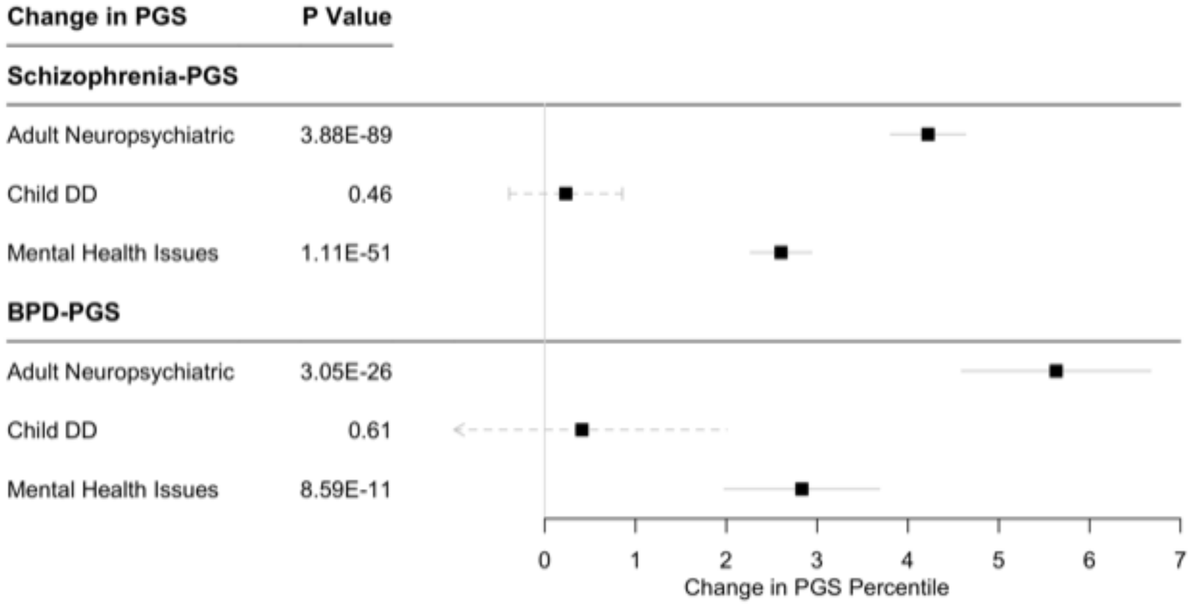

Supplementary References

1. Okbay, A. *et al.* Genome-wide association study identifies 74 loci associated with educational attainment. *Nature* **533**, 539–542 (2016).
2. Savage, J. E. *et al.* Genome-wide association meta-analysis in 269,867 individuals identifies new genetic and functional links to intelligence. *Nat. Genet.* **50**, 912–919 (2018)
3. Genç, E. *et al.* Polygenic Scores for Cognitive Abilities and Their Association with Different Aspects of General Intelligence—A Deep Phenotyping Approach. *Mol. Neurobiol.* **58**, 4145–4156 (2021).
