# Supplementary material for: Genetic modifiers of rare variants in monogenic developmental disorder loci
Source: Nat Genet. 2024 Apr 18;56(5):861–8. doi: 10.1038/s41588-024-01710-0 (PMC11096126; doi:10.1038/s41588-024-01710-0)
Supplement: Supplementary file 4 — STATA scripts. [file 41588_2024_1710_MOESM4_ESM.docx]

All analysis scripts in STATA

### Variant Burden ###

generate one_variant = 1 if variant_burden == 1

generate two_variant = 1 if variant_burden == 2

generate three_variant = 1 if variant_burden == 3

generate three_plus_variant = 1 if variant_burden == 3

replace three_plus_variant = 1 if variant_burden == 4

replace three_plus_variant = 1 if variant_burden == 5

foreach var of varlist one_variant two_variant three_variant three_plus_variant {

replace `var' = 0 if `var' != 1

}

#separating out the variant carriers into quintiles, and by how many variants they carry

generate one_variant_q1edu = 1 if one_variant == 1 & edu_quintile == 1

generate one_variant_q2edu = 1 if one_variant == 1 & edu_quintile == 2

generate one_variant_q3edu = 1 if one_variant == 1 & edu_quintile == 3

generate one_variant_q4edu = 1 if one_variant == 1 & edu_quintile == 4

generate one_variant_q5edu = 1 if one_variant == 1 & edu_quintile == 5

generate two_variant_q1edu = 1 if two_variant == 1 & edu_quintile == 1

generate two_variant_q2edu = 1 if two_variant == 1 & edu_quintile == 2

generate two_variant_q3edu = 1 if two_variant == 1 & edu_quintile == 3

generate two_variant_q4edu = 1 if two_variant == 1 & edu_quintile == 4

generate two_variant_q5edu = 1 if two_variant == 1 & edu_quintile == 5

generate three_variant_q1edu = 1 if three_variant == 1 & edu_quintile == 1

generate three_variant_q2edu = 1 if three_variant == 1 & edu_quintile == 2

generate three_variant_q3edu = 1 if three_variant == 1 & edu_quintile == 3

generate three_variant_q4edu = 1 if three_variant == 1 & edu_quintile == 4

generate three_variant_q5edu = 1 if three_variant == 1 & edu_quintile == 5

generate three_plus_variant_q1edu = 1 if three_plus_variant == 1 & edu_quintile == 1

generate three_plus_variant_q2edu = 1 if three_plus_variant == 1 & edu_quintile == 2

generate three_plus_variant_q3edu = 1 if three_plus_variant == 1 & edu_quintile == 3

generate three_plus_variant_q4edu = 1 if three_plus_variant == 1 & edu_quintile == 4

generate three_plus_variant_q5edu = 1 if three_plus_variant == 1 & edu_quintile == 5

#replacing the values with zero if individual does not fall within a group:

foreach var of varlist one_variant_q1edu one_variant_q2edu one_variant_q3edu one_variant_q4edu one_variant_q5edu two_variant_q1edu two_variant_q2edu two_variant_q3edu two_variant_q4edu two_variant_q5edu three_variant_q1edu three_variant_q2edu three_variant_q3edu three_variant_q4edu three_variant_q5edu three_plus_variant_q1edu three_plus_variant_q2edu three_plus_variant_q3edu three_plus_variant_q4edu three_plus_variant_q5edu {

replace `var' = 0 if `var' != 1

}

#linear regression for each of the tested groups, against the Q3 group who don’t carry a variant (use_these category)

file open myfile using "/file_path", write append

set more off

#traits tested against:

foreach var of varlist income tdi fluid_intelligence edu_yrs age_education reaction_time_raw_sin pairs_test height numeric_memory_raw_sin {

foreach vartwo of varlist no_variant one_variant two_variant three_variant three_plus_variant one_variant_q1edu one_variant_q2edu one_variant_q3edu one_variant_q4edu one_variant_q5edu two_variant_q1edu two_variant_q2edu two_variant_q3edu two_variant_q4edu two_variant_q5edu three_variant_q1edu three_variant_q2edu three_variant_q3edu three_variant_q4edu three_variant_q5edu three_plus_variant_q1edu three_plus_variant_q2edu three_plus_variant_q3edu three_plus_variant_q4edu three_plus_variant_q5edu {

#control for centre, sex, age, 40 pcs (all variables starting with pcs), running regression on all the variable groups created above

regress `var' `vartwo' centre sex age_base pcs* if use_these == 1 | `vartwo' == 1

file write myfile "variant_" "`var'_" "`vartwo'" _tab (r(table)[1,1]) _tab (r(table)[2,1]) _tab (r(table)[4,1]) _tab (r(table)[5,1]) _tab (r(table)[6,1]) _n

}

}

#repeating the regression analysis for binary variables – logistic regression for these

#traits tested against:

foreach var of varlist ChildDD_all AdultDD_any mental_health_new unable_to_work parent_never degree employed {

foreach vartwo of varlist no_variant one_variant two_variant three_variant three_plus_variant one_variant_q1edu one_variant_q2edu one_variant_q3edu one_variant_q4edu one_variant_q5edu two_variant_q1edu two_variant_q2edu two_variant_q3edu two_variant_q4edu two_variant_q5edu three_variant_q1edu three_variant_q2edu three_variant_q3edu three_variant_q4edu three_variant_q5edu three_plus_variant_q1edu three_plus_variant_q2edu three_plus_variant_q3edu three_plus_variant_q4edu three_plus_variant_q5edu {

logistic `var' `vartwo' centre sex age_base pcs* if use_these == 1 | `vartwo' == 1

file write myfile "edu_24_" "`var'_" "`vartwo'" _tab (r(table)[1,1]) _tab (r(table)[2,1]) _tab (r(table)[4,1]) _tab (r(table)[5,1]) _tab (r(table)[6,1]) _n

}

}

#save and quit

file close myfile

### Deviators ###

#creating groups of individuals who deviate from predicted phenotype

#low fluid intelligence and educational attainment pgs in tenth decile

generate pgs_deviator = 1 if fluid_intelligence == 1 & edu_decile == 10

replace pgs_deviator = 1 if fluid_intelligence == 0 & edu_decile == 10

#control group as individuals who have top decile edu pgs but didn’t score 0,1 on fluid intelligence test

replace pgs_deviator = 0 if pgs_deviator != 1 & edu_decile == 10

file open myfile using "/file_path", write append

set more off

#logistic regression run against individuals who fall in the above group, and various variant #carrier groups

foreach var of varlist any_variant lof cnv_del cnv_dup missense no_variant {

foreach vartwo of varlist pgs_deviator {

logistic `var' `vartwo' centre sex age_base pcs*

file write myfile "odds_ratio_vs_top_decile_" "`var'_" "`vartwo'" _tab (r(table)[1,1]) _tab (r(table)[2,1]) _tab (r(table)[4,1]) _tab (r(table)[5,1]) _tab (r(table)[6,1]) _n

}

}

file close myfile

### Diagnosis among non-carriers ###

file open myfile using "/slade/home/rk417/15_08/results/diagnosis_vs_variant.results.txt", write append

set more off

#linear regression of educational attainment pgs score against the tested traits

foreach var of varlist edu_percentile {

foreach vartwo of varlist mental_health_bk ChildDD_any AdultDD_any {

regress `var' `vartwo' centre sex age_base pcs* if any_variant != 1

file write myfile "change_in_pgs_among_non_carriers_" "`var'_" "`vartwo'" _tab (r(table)[1,1]) _tab (r(table)[2,1]) _tab (r(table)[4,1]) _tab (r(table)[5,1]) _tab (r(table)[6,1]) _n

}

}

file close myfile
